# Factors associated with prescribing costs: analysis of a nationwide administrative database

**DOI:** 10.1186/s12962-018-0091-1

**Published:** 2018-02-08

**Authors:** O. Hirsch, M. Schulz, M. Erhart, N. Donner-Banzhoff

**Affiliations:** 1 Department of General Practice/Family Medicine, Philipps University Marburg, Karl-von-Frisch-Str. 4, 35043 Marburg, Germany; 2 Central Research Institute of Ambulatory Health Care in Germany (ZI), Herbert-Lewin-Platz 3, 10623 Berlin, Germany

**Keywords:** Drug prescriptions, Drug costs, Ambulatory care, Regression analyses

## Abstract

**Objective:**

All health care systems in the world struggle with rising costs for drugs. We sought to explore factors impacting on prescribing costs in a nationwide database of ambulatory care in Germany. Factors identified by this research can be used for adjustment in future profiling efforts.

**Methods:**

We analysed nationwide prescription data of physicians having contractual relationships with statutory health insurance funds in 2014. Predictor and outcome variables were aggregated at the practice level. We performed analyses separately for primary care and specialties of cardiology, gastroenterology, neurology and psychiatry, pulmology as well as oncology and haematology. Bivariate robust regressions and Spearman rank correlations were computed in order to find meaningful predictors for our outcome variable prescription costs per patient.

**Results:**

Median age of patients and proportion of DDD issued were substantial predictors for prescription costs per patient in Primary Care, Cardiology, and Pulmology with explained variances between 41 and 61%. In Neurology and Psychiatry only proportion of patients with polypharmacy ≥ 2 quarters was a significant predictor for prescription costs per patient, explaining 20% of the variance. For gastroenterologists, oncologists and haematologists no stable models could be established.

**Conclusions:**

Any analysis of prescribing behaviour must take the degree into account to which an individual physician or practice is responsible for prescribing patients’ medication. Proportion of prescriptions/DDDs is an essential confounder for future studies of drug prescribing.

**Electronic supplementary material:**

The online version of this article (10.1186/s12962-018-0091-1) contains supplementary material, which is available to authorized users.

## Background

Without exception, all health care systems in the world struggle with rising costs for drugs. Part of the increase in prescribing budgets is regarded as inevitable and legitimate. Among possible explanations of this kind are the availability of effective novel drugs, which are usually more expensive than their predecessors, demographic changes, such as aging populations, and the identification of hitherto unknown risk states which are amenable to pharmacological intervention, such as elevated cardiovascular risk or chronic conditions between symptomatic disease episodes [1].

While the prescription of evidence-based treatments for appropriate indications is regarded as rational, there are factors influencing physicians in other directions. Prescribers’ lack of knowledge, pressure from pharmaceutical industry, biased information in professional media, unrealistic expectations from patients, to name just a few, partially interacting factors, may lead to inappropriate prescribing [2, 3].

A large variety of strategies have been developed to identify inadequate prescribing, to improve the quality of prescribing and to contain cost [4–6]. Among these are several which imply the monitoring of administrative data in order to identify prescriptions or providers not complying with the standard of rational prescribing. This procedure is called profiling and is used to monitor a wide range of processes and outcomes relevant to health and health care [7].

One of the main challenges of profiling in health care is case mix. A hospital may have higher costs per patient and higher mortality rates than a neighboring hospital. This, however, can be perfectly legitimate as long as the outcomes can be explained by sicker patients being treated than in comparison facilities. Only if legitimate factors, such as age or morbidity, are adjusted for, can profiling with regard to costs be expected to identify providers truly in need of remedial action or another intervention with the aim of improving their prescribing [8].

Age of the patient has been shown to lead to higher prescribing costs [9]. In several regions in Italy, 56% of total prescribing costs were spent for patients over 65 years. On average, prescribing costs for a 75-year-old male were 12 times higher than for a 25–34-year-old male, and prescribing costs for a 75-year-old female were 8 times higher than for a 25–34-year-old female. The study was based on prescription data from October 2004 to September 2005 from local and regional Healthcare authorities of Monza, Marche, and Basilicata with a total of about 3.2 million inhabitants [10]. An association between practice size and prescribing cost has also been shown previously [11, 12].

If practices have certain leeway with regard to issuing a prescription themselves or referring the patient to another practice, this should be adjusted for in a profiling analysis. To our knowledge, this factor and its associations with prescribing costs have not been investigated before. Physicians often choose this option in order to reduce their prescribing cost and to avoid possible sanctions [13, 14].

In this paper, we sought to explore factors impacting on prescribing costs in a nationwide database of ambulatory care in Germany. Factors identified by this research can be used for adjustment in future profiling efforts. We performed analyses separately for primary care and specialties of cardiology, gastroenterology, neurology and psychiatry, pulmology as well as oncology and haematology.

## Methods

### Inclusion

We analysed nationwide prescription data according to § 300 section 2, Social Security Code V (AVD) of physicians having contractual relationships with statutory health insurance funds. About 90% of the German population are covered by this system. Available data included all prescriptions, excluding dentists, which were filled by patients of the statutory health insurance system in pharmacies in 2014. Since information on specialty was available only for practices with one specialty, multispecialty practices were excluded from analyses. Predictor and outcome variables were aggregated at the practice level (for details see below).

### Data sources and processing

The dependent variable for this analysis was prescription costs per patient. We divided total prescription costs of each practice by the number of patients with prescriptions within the year 2014.

We investigated the following independent variables, which were also aggregated at the practice level:

- Number of patients.
- Average age of patients.
- Median age of patients.
- Proportion of pensioners.
- Proportion of female patients.
- Morbidity index: The morbidity index is based on a system of rules and an estimation formula. The system of rules consists of 34 age and gender groups and 72 diagnoses based risk groups derived from claims data. This is a hierarchical model because within a disease group only the most severe diagnosis is considered [15]. The estimation formula represents the economic evaluation of risk groups which is accomplished with a 2-year prospective model regarding the expected resource uptake. On the basis of the system of rules and the estimation formula, a morbidity index for each patient can be calculated. A value of 1.0 corresponds to the nationwide average morbidity and the nationwide average resource uptake. A value of 2.0 shows that due to age, gender, and morbidity information, twice than the average uptake in health resources would be expected. Detailed information regarding the classification model can be retrieved under the following URL: http://institut-ba.de/ba/klassifikation/km87a2015.html.
- Proportion of prescriptions issued (PPI): This reflects the degree to which a practice issues the prescriptions its patients need. This measure is low, if patients are referred to other practices, such as specialists, for their prescriptions.
- Proportion of DDD issued (PDDD): This reflects the proportion of defined daily doses (DDD) which are issued by a practice with regard to all DDDs of a patient. This measure is low, if patients are referred to other practices, such as specialists, in order to obtain their prescriptions. Because this measure truly reflects the amount of a drug prescribed, we prefer it to the measure of Proportion of prescriptions issued (PPI).
- Proportion of patients with polypharmacy: defined as having received prescriptions for 6 or more drugs per quarter.
- Proportion of patients with polypharmacy ≥ 2 quarters: defined as having received prescriptions of 6 or more drugs per quarter in a minimum of 2 quarters per year (please note that this measure is narrower than the previous polypharmacy variable).

### Statistical methods

In prescription claims data it is a well-known phenomenon that the range of values is high and the distribution of variables is far from being normal [16, 17]. Our data also deviated significantly from the normal distribution. Therefore, we applied robust and non-parametric methods for our analyses. Weighted means and weighted standard deviations were calculated for variable “Costs per patient” with the R package “Hmisc” as this variable was extremely skewed.

In order to find meaningful predictors for our outcome variable prescription costs per patient we first tested our independent variables by bivariate robust regressions using the function “lmrob” within the R package “robustbase” [18]. The rationale behind robust regression is to devaluate the influence of highly deviating data points [19]. We obtained coefficients of variance (R^2^) as measures of variance explained with ≥ .02 denoting a small, a R^2^ of ≥ .13 a medium, and a R^2^ of ≥ .26 a large effect [20]. We included those predictors in a multivariate robust regression model which had medium or large bivariate effects and with regards to content. Before inclusion we reviewed intercorrelations between potential predictors to avoid multicollinearity [21]. We evaluated the model by splitting the respective samples in random halves, running it in these subsamples and comparing the resulting R^2^.

Additionally, we calculated the Spearman rank correlation (Spearman’s rho) as a measure of association between single predictors and the outcome as well as between predictors themselves. This measure has been shown to be robust against outliers [22].

## Results

### Descriptive statistics

Table 1 shows descriptive statistics of the dependent variable prescription costs per patient and some of the potential predictors by the medical disciplines. The complete descriptive data for all predictors in each discipline can be found in Additional file 1: Table S1–S6. Due to data protection we had no further information available regarding the prescribing physicians or more detailed information about the practices (e.g. urban vs. rural).

**Table 1:** Descriptive statistics by discipline

Medical discipline	Mean number of patients per practice	Average age of patients	Proportion of female patients	Costs per patient in €
Primary care (n = 30,325)	Mean 1146.2 SD 586.6 Median 1072	53.4 6.6 53.7	57.8 6.0 57.3	291.69 17.07 262.93
Cardiology (n = 559)	Mean 777.4 SD 541.0 Median 628	66.6 3.0 67.0	48.8 6.6 48.0	215.67 14.69 149.63
Gastroenterology (n = 485)	Mean 612.2 SD 594.8 Median 487	54.7 5.9 54.4	55.3 8.5 55.7	1104.30 33.23 790.11
Neurology and Psychiatry (n = 3080)	Mean 789.9 SD 548.5 Median 756.5	56.7 6.7 57.4	63.1 8.3 63.1	769.01 27.73 507.19
Pulmology (n = 642)	Mean 1760.9 SD 1063.5 Median 1642	57.0 4.6 57.0	55.7 6.5 56.3	372.01 19.29 324.62
Oncology and Haematology (n = 303)	Mean 447.3 SD 378.3 Median 368	64.5 4.6 65.6	55.1 11.0 55.6	9181.15 95.81 9384.36

Prescribing costs were highest in Haematology & Oncology, followed by Gastroenterology. In Primary Care, costs per patient were lowest. The highest mean number of patients with prescriptions was found in Pulmology but with a relatively high standard deviation, followed by Primary Care, and Oncology & Haematology having the least mean number of patients with prescriptions. The average age of patients was highest in Cardiology, followed by Oncology & Haematology, and lowest in Primary Care. The highest mean proportion of female patients could be observed in Neurology and Psychiatry with the other disciplines showing similar distributions except for Cardiology with a slightly lower proportion of female patients than the other disciplines.

### Factors associated with prescribing costs

#### Primary care

Table 2 displays the results of the bivariate robust regression analyses in GPs/family physicians.

**Table 2:** Prescription costs per patient—results of bivariate robust regression analyses and Spearman’s—rho coefficients in GPs/family physicians (n = 30,325)

Independent variables	R^2^ lmrob	Spearman
Practice size (number of patients)	.04	.21
Average age of patients	.30	.51
Median age of patients	.31	.53
Proportion of pensioners	.32	.53
Proportion of female patients	.03	− .15
Morbidity index	.03	.23
Proportion of prescriptions issued (PPI)	.38	.49
Proportion of DDD issued (PDDD)	.36	.47
Proportion polypharmacy patients	.34	.50
Proportion polypharmacy patients ≥ 2Q	.49	.62

Older age was associated with higher costs. As could be expected, the variables “average age of patients”, “median age of patients” and “proportion of pensioners” were highly correlated. We selected “median age of patients” for the multivariate robust regression model due to the high explained variance (R^2^ = .31, Spearman’s rho = .53). The correlation between “median age of patients” and “proportion of patients with polypharmacy ≥ 2 quarters” was also high (rho = .75). The latter was therefore not included in the final model, also because it can be influenced to a certain extent by the physicians. We further included “Proportion of DDD issued” into the model as the R^2^ signaled a large effect (R^2^ = .36, Spearman-rho = .47) and this variable more truly reflects the amount of a drug prescribed than the number of prescriptions. Practice size, mean proportion of female patients and morbidity were not associated with the outcome to a relevant degree.

The sample was randomly split in half and the model was tested in both resulting samples. The R^2^ were .54 and .537, respectively. Therefore, we calculated the model in the complete sample of GPs/family physicians which resulted in a R^2^ of .54 with both predictors “median age of patients” and “proportion of DDD issued” having p values < .001 (see Additional file 1: Table S7).

#### Cardiology

In cardiology, the association with patients’ age was much weaker than in primary care (Table 3). The proportions of prescriptions and DDDs were associated with costs to a relevant degree.

**Table 3:** Prescription costs per patient—results of bivariate robust regression analyses and Spearman’s—rho coefficients in cardiologists (n = 559)

Independent variables	R^2^ lmrob	Spearman
Number of patients	.01	.04
Average age of patients	.05	.18
Median age of patients	.07	.22
Proportion of pensioners	.07	.21
Proportion of female patients	.02	− .11
Morbidity index	.02	− .04
Proportion of prescriptions issued (PPI)	.45	.67
Proportion of DDD issued (PDDD)	.36	.60
Proportion of polypharmacy patients	.00	.01
Proportion of polypharmacy patients ≥ 2Q	.00	.13

We selected “Proportion of DDD issued” for the multivariate model as this variable is more directly related to costs than the mere number of prescriptions. Furthermore, “median age of patients” seemed to us a more appropriate predictor than “proportion of pensioners” with which it was highly correlated (rho = .91). Practice size, mean proportion of female patients, morbidity, and polypharmacy were not associated with the outcome to a relevant degree. The sample was randomly split in half and the model was tested in both resulting samples. The R^2^ were .41 and .61, respectively (see Additional file 1: Table S8, S9), showing a slight instability.

#### Gastroenterology

None of the predictors analysed resulted in relevant associations for prescribing costs in gastroenterology (Table 4). Demographic characteristics of patients were even negatively correlated and the amount of variance explained was negligible.

**Table 4:** Prescription costs per patient—results of bivariate robust regression analyses and Spearman’s—rho coefficients in gastroenterology (n = 485)

Independent variables	R^2^ lmrob	Spearman
Number of patients	.01	− .49
Average age of patients	.06	− .22
Median age of patients	.05	− .20
Proportion of pensioners	.07	− .23
Proportion of female patients	.00	− .12
Morbidity index	.00	.07
Proportion of prescriptions issued (PPI)	.03	.49
Proportion of DDD issued (PDDD)	.01	.23
Proportion of polypharmacy patients	.02	.38
Proportion of polypharmacy patients ≥ 2Q	.02	.35

The lmrob procedure measured a relatively high number of outliers (n = 104) which is suggesting that the breakdown point of the lmrob function might be exceeded and consequently the results might not be valid [18]. This could explain the discrepancies between the Spearman rho correlations and the R^2^ values in Table 4.

As potential predictors we selected “average age of patients”, “number of patients”, “proportion of prescriptions issued (PPI)”, and “proportion of polypharmacy patients ≥ 2 quarters”. After randomly splitting the sample into two halves the R^2^ values were .11 and .04. In the whole sample the resulting R^2^ was .05 with “proportion of prescriptions issued (PPI)” as the only significant predictor (p = .04). The Spearman correlation between “average age of patients” and “proportion of polypharmacy patients ≥ 2 quarters” was rho = .49. After replacing “proportion of polypharmacy patients ≥ 2 quarters” with “average age of patients”, the sample was randomly split in half and the model was tested in both resulting samples. The R^2^ were .14 and .11, respectively, the R^2^ in the whole sample increased to .12 (see Additional file 1: Table S10). “Average age of patients” emerged as a significant predictor (p = .002) but with a negative sign and “Proportion of prescriptions issued (PPI)” (p = .006). Overall, results with regard to predictors for prescribing costs of gastroenterologists are unstable and the explained variance is low due to the high number of outliers.

#### Neurology and psychiatry

Age and associated measures seemed to influence prescribing costs in neurology and psychiatry practices (Table 5).

**Table 5:** Prescription costs per patient—results of bivariate robust regression analyses and Spearman’s—rho coefficients in neurology and psychiatry (n = 3080)

Independent variables	R^2^ lmrob	Spearman
Number of patients	.14	.24
Average age of patients	.15	.39
Median age of patients	.15	.41
Proportion of pensioners	.20	.45
Proportion of female patients	.02	− .14
Morbidity index	.06	.32
Proportion of prescriptions issued (PPI)	.00	− .07
Proportion of DDD issued (PDDD)	.01	− .06
Proportion of polypharmacy patients	.15	.40
Proportion of polypharmacy patients ≥ 2Q	.20	.46

We selected “proportion of pensioners”, “morbidity index”, and “proportion of polypharmacy patients ≥ 2 quarters” as potential predictors due to their high bivariate associations (R^2^ or Spearman’s rho) with costs per patient. The correlation of “proportion of polypharmacy patients ≥ 2 quarters” with “proportion of pensioners” was rho = .84. Furthermore, the correlation of “proportion of polypharmacy patients ≥ 2 quarters” with “morbidity index” was rho = .71. The only remaining predictor in the analyses was “proportion of polypharmacy patients > 2 quarters”. The sample was randomly split in half and the model was tested in both resulting samples. The R^2^ were .21 and .19, respectively. The R^2^ for the complete sample was .20 (Table 5 and Additional file 1: Table S11) which denotes a medium effect. Practice size. mean proportion of female patients, proportion of prescriptions issued (PPI), and proportion of DDD issued (PDDD) were not associated with the outcome to a relevant degree.

#### Pulmology

We found moderate associations with age in pulmology practices (Table 6).

**Table 6:** Prescription costs per patient—results of bivariate robust regression analyses and Spearman’s—rho coefficients in pulmologists (n = 642)

Independent variables	R^2^ lmrob	Spearman
Number of patients	.02	− .09
Average age of patients	.21	.46
Median age of patients	.17	.42
Proportion of pensioners	.19	.42
Proportion of female patients	.00	− .12
Morbidity index	.05	.36
Proportion of prescriptions issued (PPI)	.28	.45
Proportion of DDD issued (PDDD)	.50	.45
Proportion of polypharmacy patients	.04	.29
Proportion of polypharmacy patients ≥ 2Q	.18	.47

The correlation between “proportion of polypharmacy patients ≥ 2 quarters” and “median age of patients” was rho = .76, the correlation between “median age of patients” and “proportion of pensioners” was rho = .99. We therefore selected “median age of patients” and “proportion of DDD issued (PDDD)” for the multivariate robust regression model. Practice size, mean proportion of female patients, morbidity, and polypharmacy were not associated with the outcome to a relevant degree. The sample was randomly split in half and the model was tested in both resulting samples. The R^2^ were .59 and .61, respectively. In the whole sample the R^2^ was relatively high with .60 (see Additional file 1: Table S12).

#### Oncology and haematology

Results of bivariate analyses in Oncology and Haematology were either unstable or had little explanatory power (Table 7).

**Table 7:** Prescription costs per patient—results of bivariate robust regression analyses and Spearman’s—rho coefficients in oncologists (n = 303)

Independent variables	R^2^ lmrob	Spearman
Number of patients	.00	− .08
Average age of patients	.06	.12
Median age of patients	Not converged	.06
Proportion of pensioners	.05	.16
Proportion of female patients	.05	− .18
Morbidity index	.04	.18
Proportion of prescriptions issued (PPI)	.30	.40
Proportion of DDD issued (PDDD)	.00	.08
Proportion of polypharmacy patients	.15	.25
Proportion of polypharmacy patients ≥ 2Q	.13	.24

The discrepancy between PPI and PDDD might be related to specifics of this field with a large number of drugs given via the parenteral route. We selected “average age of patients”, “proportion of polypharmacy patients”, and “Proportion of prescriptions issued (PPI)” as predictors for a multivariate robust regression model. After randomly splitting the sample into two halves, the R^2^ were .09 and .58. This discrepancy of R^2^s suggests that the model is unstable and not an adequate representation of the data.

## Discussion

Median age of patients and proportion of DDD issued (PDDD) were substantial predictors for prescription costs per patient in Primary Care, Cardiology, and Pulmology with explained variances between 41 and 61%. In Neurology and Psychiatry only proportion of patients with polypharmacy ≥ 2 quarters was a significant predictor for prescription costs per patient, explaining 20% of the variance. For gastroenterologists, oncologists and haematologists no stable models could be established.

Variables denoting age and proportion of prescriptions issued were most frequently shown to have an impact on prescribing costs. Interestingly, with the exception of Neurology and Psychiatry, the morbidity index showed only weak or no associations with prescribing costs. Specialties varied in a characteristic fashion with Oncology and Haematology having the highest and primary care the lowest prescribing costs.

In the medical disciplines in our study, an increase of 1 year in the mean median age of patients meant an increase of about 7 Euros in prescription costs per patient. This finding of higher costs with increasing age is supported by other studies. They differ from our study in that the unit of observation is the individual patient but the cluster structure of the data is ignored. A Spanish study found a large variability between age groups regarding prescription costs [9]. Sleator found increasing prescription rates with increasing age except for the youngest age group 0–4 years, where costs were high [23]. The author argues for a differential weighting system in different age groups as the variability of prescribing costs between them is considerably high [24]. Age-sex standardised weightings were able to explain only 25% of the variation in prescribing costs among patients of general practitioners [10]. Consequently, other unexplained variables in practice populations or physician characteristics must be present here. Overall medication use equally increased with age in men and women but the use of specific drug classes differed significantly by gender [25]. Women were more likely to use antidepressants, antianxiety, and pain medications while men were using cardiovascular medications at an earlier age than women [9, 25]. These results were not confirmed by our data as we did not find a significant contribution of gender to prescription costs per patient in any of the disciplines. Nevertheless, these negative findings support the case for differential analyses at the individual (patient) level which would probably reflect the effect of gender on the prescription of specific drug classes. Analysing prescription data on individual patient level Omar et al. found that individual patient morbidity was the strongest predictor to explain variation in prescribing [8]. Age and sex were only able to explain 10% of the variation in prescribing, after adding diagnoses based morbidity, the explained variance increased to 80%. This is in contrast to our finding on aggregate level where morbidity was not associated with either proportion of prescriptions issued (PPI) nor with proportion of DDD issued (PDDD).

A Finish study found a very skewed prescription cost distribution on patient level with 5% of the patients accounting for about half of the costs and with about one-fourth of them being 75 over years old. Therefore, the authors argue for more patient-specific cost-containment methods [26]. The skewness also present in our data was related to practices, especially in cardiology, gastroenterology, neurology and psychiatry, and pulmology. In other words, most practices were on the left side (lower cost) of the distribution, but a smaller number of practices had high and very high prescribing costs. Specific patient and/or practice subgroups are likely to be hidden in the data which can only be identified with analyses based on individual prescriptions but accommodating for clustering (patient, practice) [9–11].

The proportion of DDD issued was another important cost predictor in our data for Primary Care, Cardiology, and Pulmology. This is perhaps more relevant for settings or health care systems where not clear gatekeeping is established. In Germany, especially the chronically ill are to a varying degree cared for by community specialists. Any analysis of prescribing behaviour must take the degree into account to which an individual physician or practice is responsible for prescribing patients’ medication.

In Germany, measures to control prescribing costs have been in place for more than 30 years [13, 14]. Practices are systematically checked whether their prescribing costs per patient exceed those of their peers. If higher costs cannot be explained by case mix, practices are asked to provide justification for their prescribing and, if explanations are not regarded as satisfactory, may even suffer financial sanctions. Although this has happened only very rarely recently, physicians still feel under pressure to contain their prescribing costs, and shift (refer) patients to other physicians in an attempt to reduce their individual prescribing cost and thus to avoid sanctions.

### Limitations

The prescribing behavior of physicians is highly complex and determined by a large number of factors. Only a small part of these was represented in our data set. Moreover, we analysed physician behavior and patients’ characteristics only aggregated at the practice level. Analyses of individual prescriptions while accounting for clustering would have been desirable but were not possible due to confidentially regulation. Prescription costs per case would have been desirable in order to improve comparability between the different medical disciplines but this variable was not available. For morbidity, only generic (multimorbidity) measure was available.

We had prescription data available for drugs that were completely reimbursed by the statutory health insurances in Germany. No data were available for over-the-counter (OTC) drugs, drugs prescribed to in-patients in hospitals or drugs reimbursed by private health insurances.

Variables influencing the prescription process like knowledge, professional norms, and patients’ expectations regarding prescriptions could not be investigated. Characteristics of prescribing physicians were not represented in our data base. Some of these such as age, gender, working in group practice have previously been shown to influence prescribing costs. Younger physicians certified in the past 10 years, female physicians, and those practising in a group issued more prescriptions than their respective counterparts and thereby causing higher costs. There were also strong geographical effects [11, 12].

## Conclusion

Our study shows the relevance of a factor impacting on prescribing costs which has not been acknowledged previously. Once physicians have some leeway deciding whether to prescribe a drug themselves or refer the patient to another practice, the proportion of prescriptions/DDDs must be accounted for when the volume and/or appropriateness of physicians’ prescribing is investigated. Shifting patients to other providers may be a deliberate gaming strategy used by physicians to avoid sanctions regarding one’s prescribing. Proportion of prescriptions/DDDs is thus an essential confounder for future studies of drug prescribing.

## Additional file

**Additional file 1: Table S1.** Descriptive statististics (mean, standard deviation, and median) regarding the potential predictors median age of patients, proportion of pensioners, morbidity index, proportion of prescriptions issued (PPI), proportion of DDD issued (PDDD), proportion of polypharmacy patients, and proportion of polypharmacy patients in ≥ 2 quarters in GPs and family physicians (n=30,325). **Table S2.** Descriptive statististics (mean, standard deviation, and median) regarding the potential predictors median age of patients, proportion of pensioners, morbidity index, proportion of prescriptions issued (PPI), proportion of DDD issued (PDDD), proportion of polypharmacy patients, and proportion of polypharmacy patients in ≥ 2 quarters in cardiologists (n=559). **Table S3.** Descriptive statististics (mean, standard deviation, and median) regarding the potential predictors median age of patients, proportion of pensioners, morbidity index, proportion of prescriptions issued (PPI), proportion of DDD issued (PDDD), proportion of polypharmacy patients, and proportion of polypharmacy patients in ≥ 2 quarters in gastroenterologists (n=485). **Table S4.** Descriptive statististics (mean, standard deviation, and median) regarding the potential predictors median age of patients, proportion of pensioners, morbidity index, proportion of prescriptions issued (PPI), proportion of DDD issued (PDDD), proportion of polypharmacy patients, and proportion of polypharmacy patients in ≥ 2 quarters in neurologists and psychiatrists (n=3080). **Table S5.** Descriptive statististics (mean, standard deviation, and median) regarding the potential predictors median age of patients, proportion of pensioners, morbidity index, proportion of prescriptions issued (PPI), proportion of DDD issued (PDDD), proportion of polypharmacy patients, and proportion of polypharmacy patients in ≥ 2 quarters in pulmologists (n=642). **Table S6.** Descriptive statististics (mean, standard deviation, and median) regarding the potential predictors median age of patients, proportion of pensioners, morbidity index, proportion of prescriptions issued (PPI), proportion of DDD issued (PDDD), proportion of polypharmacy patients, and proportion of polypharmacy patients in ≥ 2 quarters in oncologists and haematologists (n=303). **Table S7.** Results of the multivariate robust regression model (R^2^ =.54) with dependent variable „costs per patient“ in the complete sample of GPs/family physicians (n=30,325). **Table S8.** Results of the multivariate model (R^2^ =.41) with dependent variable „costs per patient“ in cardiologists (sample 1, n=280). **Table S9.** Results of the multivariate model (R^2^ =.61) with dependent variable „costs per patient“ in cardiologists (sample 2, n=279). **Table S10.** Results of the multivariate model (R^2^ =.12) with dependent variable „costs per patient“ in gastroenterologists (n=485). **Table S11.** Results of the multivariate model (R^2^ =.20) with dependent variable „costs per patient“ in neurologists and psychiatrists (n=3080). **Table S12.** Results of the multivariate model (R^2^ =.60) with dependent variable „costs per patient“ in pulmologists (n=642).
