# Supplementary material for: Factors associated with prescribing costs: analysis of a nationwide administrative database
Source: Cost Eff Resour Alloc. 2018 Feb 8;16:5. doi: 10.1186/s12962-018-0091-1 (PMC5806480; doi:10.1186/s12962-018-0091-1)
Supplement: Supplementary file 1 — Additional file 1: Table S1. Descriptive statististics (mean, standard deviation, and median) regarding the potential predictors median age of patients, proportion of pensioners, morbidity index, proportion of prescriptions issued (PPI), proportion of DDD issued (PDDD), proportion of polypharmacy patients, and proportion of polypharmacy patients in ≥ 2 quarters in GPs and family physicians (n=30,325). Table S2. Descriptive statististics (mean, standard deviation, and median) regarding the potential predictors median age of patients, proportion of pensioners, morbidity index, proportion of prescriptions issued (PPI), proportion of DDD issued (PDDD), proportion of polypharmacy patients, and proportion of polypharmacy patients in ≥ 2 quarters in cardiologists (n=559). Table S3. Descriptive statististics (mean, standard deviation, and median) regarding the potential predictors median age of patients, proportion of pensioners, morbidity index, proportion of prescriptions issued (PPI), proportion of DDD issued (PDDD), proportion of polypharmacy patients, and proportion of polypharmacy patients in ≥ 2 quarters in gastroenterologists (n=485). Table S4. Descriptive statististics (mean, standard deviation, and median) regarding the potential predictors median age of patients, proportion of pensioners, morbidity index, proportion of prescriptions issued (PPI), proportion of DDD issued (PDDD), proportion of polypharmacy patients, and proportion of polypharmacy patients in ≥ 2 quarters in neurologists and psychiatrists (n=3080). Table S5. Descriptive statististics (mean, standard deviation, and median) regarding the potential predictors median age of patients, proportion of pensioners, morbidity index, proportion of prescriptions issued (PPI), proportion of DDD issued (PDDD), proportion of polypharmacy patients, and proportion of polypharmacy patients in ≥ 2 quarters in pulmologists (n=642). Table S6. Descriptive statististics (mean, standard deviation, and median) regarding the [file 12962_2018_91_MOESM1_ESM.docx]

**Factors associated with prescribing costs. Analysis of a nationwide administrative database**

**The European Journal of Health Economics**

**Hirsch, O.^1^, Schulz, M.^2^, Erhart, M.^2^, Donner-Banzhoff, N.^1^**

**^1^ Department of General Practice/Family Medicine, Philipps University Marburg**

**^2^ Zentralinstitut für die kassenärztliche Versorgung in Deutschland (Zi), Berlin**

**Corresponding author:**

Dr.Oliver Hirsch

Department of General Practice/Family Medicine

Philipps University Marburg

Karl-von-Frisch-Str. 4

35043 Marburg

Germany

Tel.: 06421/2865124

Fax: 06421/2865121

E-Mail: [oliver.hirsch@staff.uni-marburg.de](mailto:oliver.hirsch@staff.uni-marburg.de)

## Additional Material

Table S1. Descriptive statististics (mean, standard deviation, and median) regarding the potential predictors median age of patients, proportion of pensioners, morbidity index, proportion of prescriptions issued (PPI), proportion of DDD issued (PDDD), proportion of polypharmacy patients, and proportion of polypharmacy patients in ≥ 2 quarters in GPs and family physicians (n=30325).

| Median age of patients | Mean 55.0  SD 7.5  Median 55.0 |
| --- | --- |
| Proportion of pensioners | Mean 40.7  SD 12.4  Median 40.5 |
| Morbidity index | Mean 1.53  SD 0.26  Median 1.49 |
| Proportion of prescriptions issued (PPI) | Mean 0.58  SD 0.12  Median 0.59 |
| Proportion of DDD issued (PDDD) | Mean 0.63  SD 0.13  Median 0.66 |
| Proportion of polypharmacy patients | Mean 49.1  SD 8.0  Median 48.7 |
| Proportion of polypharmacy patients ≥2Q | Mean 23.2  SD 7.7  Median 22.4 |

Table S2. Descriptive statististics (mean, standard deviation, and median) regarding the potential predictors median age of patients, proportion of pensioners, morbidity index, proportion of prescriptions issued (PPI), proportion of DDD issued (PDDD), proportion of polypharmacy patients, and proportion of polypharmacy patients in ≥ 2 quarters in cardiologists (n=559).

| Median age of patients | Mean 69.1  SD 3.4  Median 70.0 |
| --- | --- |
| Proportion of pensioners | Mean 70.8  SD 8.0  Median 71.7 |
| Morbidity index | Mean 2.09  SD 0.24  Median 2.06 |
| Proportion of prescriptions issued (PPI) | Mean 0.14  SD 0.08  Median 0.12 |
| Proportion of DDD issued (PDDD) | Mean 0.16  SD 0.10  Median 0.13 |
| Proportion of polypharmacy patients | Mean 80.9  SD 6.9  Median 82.3 |
| Proportion of polypharmacy patients ≥2Q | Mean 52.9  SD 8.9  Median 54.0 |

Table S3. Descriptive statististics (mean, standard deviation, and median) regarding the potential predictors median age of patients, proportion of pensioners, morbidity index, proportion of prescriptions issued (PPI), proportion of DDD issued (PDDD), proportion of polypharmacy patients, and proportion of polypharmacy patients in ≥ 2 quarters in gastroenterologists (n=485).

| Median age of patients | Mean 55.8  SD 7.0  Median 56.0 |
| --- | --- |
| Proportion of pensioners | Mean 40.4  SD 14.4  Median 39.7 |
| Morbidity index | Mean 1.79  SD 0.35  Median 1.71 |
| Proportion of prescriptions issued (PPI) | Mean 0.21  SD 0.11  Median 0.18 |
| Proportion of DDD issued (PDDD) | Mean 0.19  SD 0.11  Median 0.17 |
| Proportion of polypharmacy patients | Mean 65.9  SD 9.9  Median 64.1 |
| Proportion of polypharmacy patients ≥2Q | Mean 31.7  SD 12.2  Median 28.6 |

Table S4. Descriptive statististics (mean, standard deviation, and median) regarding the potential predictors median age of patients, proportion of pensioners, morbidity index, proportion of prescriptions issued (PPI), proportion of DDD issued (PDDD), proportion of polypharmacy patients, and proportion of polypharmacy patients in ≥ 2 quarters in neurologists and psychiatrists (n=3080).

| Median age of patients | Mean 57.4  SD 7.6  Median 57.0 |
| --- | --- |
| Proportion of pensioners | Mean 40.9  SD 16.7  Median 43.0 |
| Morbidity index | Mean 1.97  SD 0.31  Median 1.94 |
| Proportion of prescriptions issued (PPI) | Mean 0.26  SD 0.08  Median 0.25 |
| Proportion of DDD issued (PDDD) | Mean 0.24  SD 0.09  Median 0.23 |
| Proportion of polypharmacy patients | Mean 67.4  SD 9.4  Median 68.9 |
| Proportion of polypharmacy patients ≥2Q | Mean 38.5  SD 10.8  Median 39.4 |

Table S5. Descriptive statististics (mean, standard deviation, and median) regarding the potential predictors median age of patients, proportion of pensioners, morbidity index, proportion of prescriptions issued (PPI), proportion of DDD issued (PDDD), proportion of polypharmacy patients, and proportion of polypharmacy patients in ≥ 2 quarters in pulmologists (n=642).

| Median age of patients | Mean 59.7  SD 4.9  Median 60.0 |
| --- | --- |
| Proportion of pensioners | Mean 49.3  SD 9.8  Median 48.6 |
| Morbidity index | Mean 1.97  SD 0.30  Median 1.90 |
| Proportion of prescriptions issued (PPI) | Mean 0.18  SD 0.06  Median 0.17 |
| Proportion of DDD issued (PDDD) | Mean 0.16  SD 0.06  Median 0.16 |
| Proportion of polypharmacy patients | Mean 77.0  SD 6.8  Median 77.0 |
| Proportion of polypharmacy patients ≥2Q | Mean 44.9  SD 9.5  Median 44.2 |

Table S6. Descriptive statististics (mean, standard deviation, and median) regarding the potential predictors median age of patients, proportion of pensioners, morbidity index, proportion of prescriptions issued (PPI), proportion of DDD issued (PDDD), proportion of polypharmacy patients, and proportion of polypharmacy patients in ≥ 2 quarters in oncologists and haematologists (n=303).

| Median age of patients | Mean 66.9  SD 5.5  Median 68.0 |
| --- | --- |
| Proportion of pensioners | Mean 65.2  SD 11.9  Median 67.9 |
| Morbidity index | Mean 2.68  SD 0.41  Median 2.65 |
| Proportion of prescriptions issued (PPI) | Mean 0.40  SD 0.11  Median 0.40 |
| Proportion of DDD issued (PDDD) | Mean 0.20  SD 0.11  Median 0.17 |
| Proportion of polypharmacy patients | Mean 84.4  SD 8.3  Median 86.1 |
| Proportion of polypharmacy patients ≥2Q | Mean 55.9  SD 10.3  Median 56.8 |

Table S7. Results of the multivariate robust regression model (R^2^ =.54) with dependent variable „costs per patient“ in the complete sample of GPs/family physicians (n=30325).

|  | Estimate | Std. error | t value | p |
| --- | --- | --- | --- | --- |
| Intercept | -229.2 | 3.38 | -67.73 | <.001 |
| Median age patients | 5.10 | 0.06 | 87.47 | <.001 |
| Proportion of DDD issued | 328.81 | 2.86 | 114.80 | <.001 |

Table S8. Results of the multivariate model (R^2^ =.41) with dependent variable „costs per patient“ in cardiologists (sample 1, n=280).

|  | Estimate | Std. error | t value | p |
| --- | --- | --- | --- | --- |
| Intercept | -412.0 | 74.61 | -5.52 | <.001 |
| Median age patients | 7.03 | 1.11 | 6.32 | <.001 |
| Proportion of DDD issued | 468.78 | 69.42 | 6.75 | <.001 |

Table S9. Results of the multivariate model (R^2^ =.61) with dependent variable „costs per patient“ in cardiologists (sample 2, n=279).

|  | Estimate | Std. error | t value | p |
| --- | --- | --- | --- | --- |
| Intercept | -459.3 | 72.67 | -6.32 | <.001 |
| Median age patients | 7.49 | 1.07 | 6.98 | <.001 |
| Proportion of DDD issued | 602.44 | 47.42 | 12.71 | <.001 |

Table S10. Results of the multivariate model (R^2^ =.12) with dependent variable „costs per patient“ in gastroenterologists (n=485).

|  | Estimate | Std. error | t value | p |
| --- | --- | --- | --- | --- |
| Intercept | 3007.8 | 813.7 | 3.70 | <.001 |
| Average age patients | -46.55 | 14.56 | -3.20 | .002 |
| Proportion of prescriptions issued | 1703.24 | 613.65 | 2.78 | .006 |
| Number of patients | -0.08 | 0.06 | -1.36 | .17 |

Table S11. Results of the multivariate model (R^2^ =.20) with dependent variable „costs per patient“ in neurologists and psychiatrists (n=3080).

|  | Estimate | Std. error | t value | p |
| --- | --- | --- | --- | --- |
| Intercept | 61.6 | 16.2 | 3.80 | <.001 |
| Proportion of polypharmacy patients ≥2 quarters | 11.61 | 0.47 | 24.65 | <.001 |

Table S12. Results of the multivariate model (R^2^ =.60) with dependent variable „costs per patient“ in pulmologists (n=642).

|  | Estimate | Std. error | t value | p |
| --- | --- | --- | --- | --- |
| Intercept | -419.4 | 47.5 | -8.82 | <.001 |
| Median age of patients | 8.02 | 0.80 | 10.09 | <.001 |
| Proportion of DDD issued | 1620.58 | 77.37 | 20.95 | <.001 |
